# A novel role of ultrasonography in identification of the tract of abdominal stab injury

**DOI:** 10.1186/2036-7902-6-S1-A30

**Published:** 2014-01-31

**Authors:** Chih-Yin Ku, Chih-Jung Chang, Chun-Yen Huang, Jen-Tang Sun, Heng-Fu Lin, Mau-Sheng Lin, Kuang-Chau Tsai

**Affiliations:** 1 Department of Emergency Medicine, Far Eastern Memorial Hospital, New Taipei City, Taiwan; 2 Department of General Surgery, Far Eastern Memorial Hospital, New Taipei City, Taiwan

## Background

Abdominal stab injury is potential life-threatening injuries. Operation should be considered if wound violate the peritoneal layer and/or complicated with hemodynamic compromise, peritonitis, impalement, or evisceration. It is well known there are several diagnostic tools to evaluate the necessity of operation for a patient with abdominal stab injury. Despite numerous diagnostic tools, including local wound exploration, CT, diagnostic peritoneal lavage, or FAST, were currently performed in emergent department neither of them has satisfying sensitivity and specificity. Abdominal wall ultrasonography has been used many abdominal wall disease, like hernia, abdominal wall hematoma. We used abdominal wall ultrasonography for diagnosis of abdominal wall stabbing injury

## Objective

Feasibility of abdominal wall ultrasonography for diagnosis of abdominal wall stabbing injury

## Patients and methods

A 76-year-old man visited our emergent department due to multiple anterior abdominal stab wounds for suicide.(Figure 1) His initially vital signs were relatively stable( The initial blood pressure was 203/110 mm Hg, the pulse 83 beats per minute, and the respiratory rate 20 breaths per minute). Physical examination revealed multiple stabbing wound with bleeding, local tender over wound without rebounding tenderness. Laboratory finding is nothing particular. Patient received local wound exploration by senior surgical resident but inconclusive. Focused Assessment of Sonography for Trauma also showed negative result. Therefore, we perform the abdominal wall ulrasonography and revealed an apparent penetrating tract through the abdominal wall and disruption of peritoneal layer.(figure 2 and video 1) We use Toshiba Nemio( 3.75mHz abdominal probe) in sterile method from start to finish. Computed tomography also revealed penetrating injury of abdominal wall and no obvious free air.(figure 3) Finally, the patient underwent the laparoscopic surgery and revealed four abdominal wall stab wounds at the mild abdomen near the umbilicus and the epigastric area(fiugre 4), perforation of transverse colon. Then he received laparoscopic repair of abdominal wall injury and colostomy. Patient was discharged smoothly after surgery.

## Results

We performed the abdominal wall ultrasonography to evaluate the stab wound and successfully diagnosed the wound penetrating peritoneal layering. Moreover, in comparison with other diagnostic tools, abdominal wall ultrasonography can provide additional advantages, such as rapid, painless, non-invasion, non-radiation and more detail of wound tract structure. By using the ultrasonography, which could follow the tract of abdominal stab injury (the direct sign) and hematoma beneath the stab wound(the indirect sign) in another case(figure 5), or positive of FAST. Abdominal wall ultrasonagraphy can help clinicians to make the decision easier in further management of abdominal stab injury.

## Conclusion

The abdominal wall ultrasonography could demonstrate the tract of stab wound, and provide more detail of wound, and help the decision making, especially in hemodynamic stable patient. Further prospective study is need for evaluating the abdominal wall ultrasonograpy in stabbing wound.

